# Steady-State Thermodynamics of a Cascaded Collision Model

**DOI:** 10.3390/e24050644

**Published:** 2022-05-03

**Authors:** Lu Li, Zhong-Xiao Man, Yun-Jie Xia

**Affiliations:** Shandong Provincial Key Laboratory of Laser Polarization and Information Technology, Department of Physics, Qufu Normal University, Qufu 273165, China; liluqfsfdx@163.com (L.L.); yjxia@qfnu.edu.cn (Y.-J.X.)

**Keywords:** collision model, quantum thermodynamics, cascaded model

## Abstract

We study the steady-state thermodynamics of a cascaded collision model where two subsystems $S_{1}$ and $S_{2}$ collide successively with an environment *R* in the cascaded fashion. We first formulate general expressions of thermodynamics quantities and identify the nonlocal forms of work and heat that result from cascaded interactions of the system with the common environment. Focusing on a concrete system of two qubits, we then show that, to be able to unidirectionally influence the thermodynamics of $S_{2}$, the former interaction of $S_{1}-R$ should not be energy conserving. We finally demonstrate that the steady-state coherence generated in the cascaded model is a kind of useful resource in extracting work, quantified by ergotropy, from the system. Our results provide a comprehensive understanding on the thermodynamics of the cascaded model and a possible way to achieve the unidirectional control on the thermodynamics process in the steady-state regime.

## 1. Introduction

Recent years have seen increasing interests in the study of quantum thermodynamics (QT) [1,2] which exploits an open quantum system [3] as a working substance to implement thermodynamic tasks. The main purposes of QT are to examine fundamental laws of classical thermodynamics in the quantum level and to reveal the influences of various quantum resources and/or quantum effects on thermodynamics processes, among others. The applications of quantum resources such as quantum coherence and correlation in QT have attracted much attention since the pioneering work of Scully et al. [4]. In Ref. [4], by conveying the atomic beam with coherence through the cavity field and interacting with the field mode for a short duration, Scully et al. have shown that the field can arrive at a larger temperature than the situation where the atoms are prepared in regular thermal states. As a result, the efficiency of the photonic Carnot engine driven by a coherent bath can outperform its classical counterpart [4]. Subsequently, the effects of quantum coherence and correlation have been applied to improve the performance of thermal machines [5,6,7,8,9,10,11,12,13,14], enhance the extraction of work [15,16,17], and increase the thermalization temperature of a quantum system [18,19,20].

Apart from specific quantum resources and quantum effects, researchers also try to explore quantum thermodynamics by engineering the couplings of a quantum system with external environments. The simultaneous couplings of several systems with a common environment lead to the noise-induced interference, which proves to be useful in enhancing the thermodynamics tasks [21,22,23,24]. Manzano et al. show that it is possible to improve the machine performance by virtue of common noise sources [22]. Albeit surrounded by a common environment, unidirectional (one-way) influence of two systems, say, $S_{1}$ and $S_{2}$, can be achieved by the so-called cascaded model [25,26,27,28,29]. In this model, $S_{1}$ and $S_{2}$ successively interact with the common environment *R* in such a way that the interaction of $S_{1}-R$ takes place first, which is then followed by $S_{2}-R$ resulting in the one-way influence of $S_{1}$ to the dynamics of $S_{2}$. The cascaded model is an efficient tool to depict the ordered interactions of individual subsystems with a common environment. For instance, in the cavity QED, the unidirectional exchange of information (energy) between a linear array of cavities and the successively injected atoms can be described by this framework [26]. The cascaded fashion of interaction is also applied in QT to study the dynamics of heat current [25,28,29]. In Ref. [25], Lorenzo et al. explore the non-Markovian dynamics of heat current and the effects of quantum correlations in dissipative cascaded systems. It turns out that, in clear contrast to the configuration with each subsystem being independently coupled to the reservoir, the heat flow under the cascaded interaction exhibits a non-exponential time behavior [25]. In these studies of the cascaded model [25,26,27,28,29], the system–environment interactions are assumed to be energy conserving so that all the energy changes are accounted for by heat without the contribution of work. In this paper, we relax the assumption of energy conservation of the system–environment interaction to make a comprehensive exploration on the thermodynamics of cascaded model involving both heat and work.

The quantum master equation (QME) is the most popular tool in the study of the dynamics of an open quantum system even with several approximations. Since QME cannot capture information about the global state of the system and environment, applications of QME in dealing with QT in some cases might lead to the occurrence of thermodynamic inconsistencies [30,31,32,33,34,35,36,37,38,39,40,41]. In Ref. [41], Levy and Kosloff have considered a model consisting of two subsystems embedded in two independent thermal reservoirs with different temperatures. It is found that the heat flows automatically from cold to hot reservoirs, i.e., the second law of thermodynamics is violated, if the local QME is used to describe the system’s dynamics [41]. A possible way to overcome this weakness is the collision model (CM) [42] where the environment is modeled as a collection of identically prepared ancillas and at each time step the system interacts/collides with a fresh ancilla. The CM has been used as an alternative tool in the study of dynamics of an open quantum system [43,44,45,46,47,48,49,50] for a long time. In particular, the CM is more efficient in the simulation of non-Markovian dynamics through several strategies, such as the introduction of either initial correlations between ancillas or ancilla–ancilla collisions between two successive system–ancilla collisions [51,52,53,54,55,56,57,58,59,60,61]. An advantage of the CM over the QME is that it can track the information of environment. In the field of QT [62,63,64,65,66,67,68,69,70,71,72,73,74], CM resolves some thermodynamics inconsistencies and makes the most fundamental definitions of thermodynamic quantities, such as heat and work, possible. It has been recognized that, in the CM, a certain amount of work should be supplied to maintain the successive collisions of the system and environment [62]. By taking the extra work cost of maintaining the successive collisions into account, the local QME is shown to comply again with thermodynamics [63]. By means of CM, the effects of non-Markovianity on the laws of thermodynamics and on the performances of thermal machines have also been studied [28,75,76,77,78,79].

In this work, we address the thermodynamics of a cascaded model where two subsystems $S_{1}$ and $S_{2}$ collide successively with an environment *R*. Thanks to the framework of CM, we can construct general formulations of thermodynamics quantities from the most fundamental definitions. Nonlocal forms of work and heat are identified as a result of cascaded interaction of the subsystems with the common environment. We then demonstrate the features of steady-state thermodynamics concentrating on a two-qubit system. It turns out that, only when the interaction of $S_{1}-R$ does not satisfy strict energy conservation, can it have a one-way effect on the thermodynamics regarding $S_{2}$. We also show that the cascaded interaction leads to steady-state coherence of the system, which can be recognized as a kind of resource in extracting useful work from the system.

## 2. The Model and Master Equation

**Figure 1:**
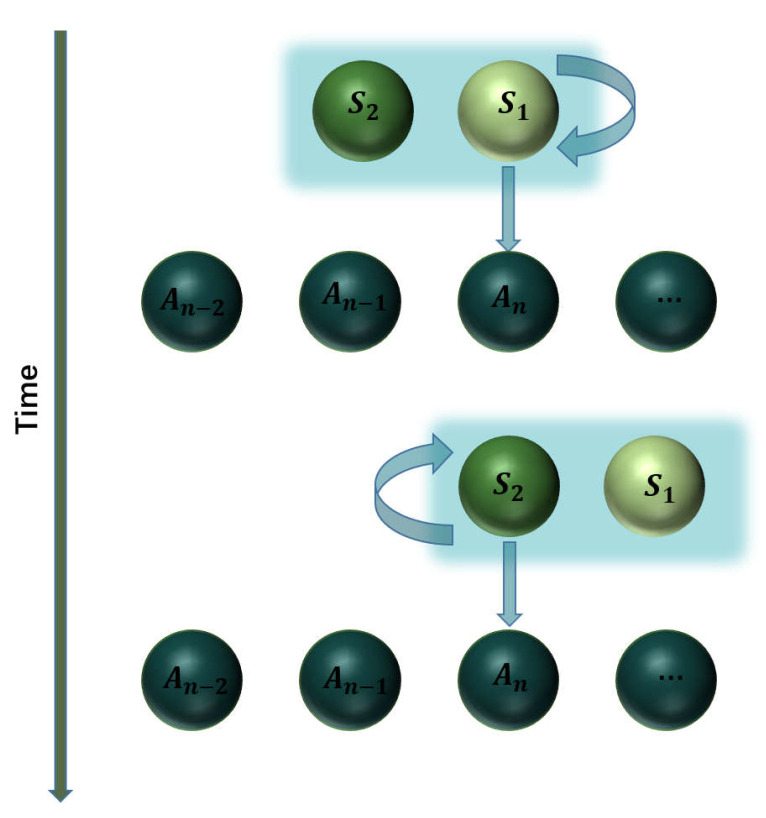
Schematic diagram of the cascaded model. The system consists of two subsystems $S_{1}$ and $S_{2}$, while the environment is modeled as a collection of ancillas with the *n*th one being labeled as $A_{n}$. In the *n*th round of the collision, $S_{1}$ collides with $A_{n}$ for a duration τ, which is then followed by another collision of $S_{2}$ with $A_{n}$ for the same duration.

We consider that the system *S* consists of two uncoupled subsystems $S_{1}$ and $S_{2}$ with the free Hamiltonian ${\hat{H}}_{S_{i}}$ (i=1,2). In terms of CM, the environment *R* is modeled as a series of identical ancillas described by the Hamiltonian ${\hat{H}}_{R}$. Note that we use ${\hat{H}}_{R}$ to represent both the environment and ancillas therein. The system–environment interactions adopt the cascaded manner in such a way that at each time step $S_{1}$ collides with an ancilla for a short duration τ, which is followed by another collision of $S_{2}$ with that ancilla for the same time τ, as depicted in Figure 1. The environment is assumed to be sufficiently large so that the system never collides twice with the same ancilla. Although there is no direct coupling between these two subsystems, the cascaded model results in unidirectional influences of $S_{1}$ to $S_{2}$ but no backward actions.

The total Hamiltonian of the system plus environment can be expressed as

(1) $${\hat{H}}_{tot}\left(t\right)={\hat{H}}_{S}+{\hat{H}}_{R}+\sum_{i=1}^{2}\lambda_{i}\left(t\right){\hat{H}}_{int}^{\left(i\right)}$$

where ${\hat{H}}_{S}=\sum_{i=1}^{2}{\hat{H}}_{S_{i}}$, ${\hat{H}}_{int}^{\left(i\right)}\equiv {\hat{V}}_{int}^{\left(i\right)}/\sqrt{\tau }$ is the interaction Hamiltonian of $S_{i}$ with *R* and we have scaled it by the interaction time τ for the convenience of taking continuous time limit although not necessary. The step function $\lambda_{i}\left(t\right)$ in Equation (1) denotes the time-dependence of the system–environment collisions and has the value 1 when $t\in \left[(n-2+i)\tau ,(n-1+i)\tau \right]$ with n≥1 the number of collisions and zero otherwise. After a round of collision, the state $\rho_{S}$ of the system at time *t* will be transformed to $\rho_{S}^{\prime }$ at time t+2τ as

(2) $$\begin{array}{c} \rho_{S}^{\prime }={\mathrm{tr}}_{R}\left\{{\hat{U}}_{2}\left(\tau \right){\hat{U}}_{1}\left(\tau \right)\rho_{S}⊗\rho_{R}^{th}{\hat{U}}_{1}^{†}\left(\tau \right){\hat{U}}_{2}^{†}\left(\tau \right)\right\}, \end{array}$$

in which ${\hat{U}}_{i}\left(\tau \right)=e^{-i\tau ({\hat{H}}_{S_{i}}+{\hat{H}}_{R}+{\hat{H}}_{int}^{\left(i\right)})}$ is the unitary time evolution operator and $\rho_{R}^{th}=e^{-\beta_{R}{\hat{H}}_{R}}/Z_{R}$ is the initial state of the environment, which has been assumed to be prepared in the thermal state at inverse temperature $\beta_{R}$ with $Z_{R}=\mathrm{tr}\left\{e^{-\beta_{R}{\hat{H}}_{R}}\right\}$ as the corresponding partition function. We set $\hbar =k_{B}=1$ here and throughout the paper. By expanding ${\hat{U}}_{i}\left(\tau \right)$ to the second order of τ, we derive the master equation governing the system’s dynamics as

(3) $$\begin{array}{ccc} {\dot{\rho }}_{S} & = & \lim_{\tau \to 0}\left(\rho_{S}^{\prime }-\rho_{S}\right)/\tau \\ & = & -i\left[{\hat{H}}_{S},\rho_{S}\right]+\sum_{i=1}^{2}L_{i}\left(\rho_{S}\right)+D_{12}\left(\rho_{S}\right), \end{array}$$

where

(4) $$L_{i}\left(\rho_{S}\right)=-\frac{1}{2}{\mathrm{tr}}_{R}\left\{\left[{\hat{V}}_{int}^{\left(i\right)},\left[{\hat{V}}_{int}^{\left(i\right)},\rho_{S}⊗\rho_{R}^{th}\right]\right]\right\}$$

stands for the local dissipation of $S_{i}$ being consistent with the situation when only $S_{i}$ exists without the involvement of other subsystems, and

(5) $$D_{12}\left(\rho_{S}\right)=-{\mathrm{tr}}_{R}\left\{\left[{\hat{V}}_{int}^{\left(2\right)},\left[{\hat{V}}_{int}^{\left(1\right)},\rho_{S}⊗\rho_{R}^{th}\right]\right]\right\}$$

characterizes the collective actions of the environment on the two subsystems owning to the cascaded interactions.

## 3. Thermodynamic Quantities of the Cascaded Model

In quantum thermodynamics, the work is generally defined as the change of energy induced by the change of the time-dependent Hamiltonian of the total system. In the CM, the successive couplings and decouplings of the system with the environment lead to the time dependence of the interaction Hamiltonian, as shown in Equation (1); therefore, the energetic cost of sustaining such successive collisions is supplied in the form of work. In a round of collisions started from *t* to t+2τ, the work performed on the system can be formulated as

(6) $$\begin{array}{ccc} \Delta W & = & {\mathrm{tr}}_{SR}\left\{{\hat{H}}_{tot}\left(t+2\tau \right)\rho_{SR}^{\prime }\right\}-{\mathrm{tr}}_{SR}\left\{{\hat{H}}_{tot}\left(t\right)\rho_{SR}\right\}\\ & = & \int_{t}^{t+2\tau }ds\frac{\partial }{\partial s}{\mathrm{tr}}_{SR}\left\{{\hat{H}}_{tot}\left(s\right)\rho_{SR}\right\}\\ & = & \int_{t}^{t+2\tau }ds{\mathrm{tr}}_{SR}\left\{\frac{\partial {\hat{H}}_{tot}\left(s\right)}{\partial s}\rho_{SR}\right\}+\int_{t}^{t+2\tau }ds{\mathrm{tr}}_{SR}\left\{{\hat{H}}_{tot}\left(s\right)\frac{\partial \rho_{SR}}{\partial s}\right\}, \end{array}$$

in which $\rho_{SR}=\rho_{S}⊗\rho_{R}^{th}$ and $\rho_{SR}^{\prime }={\hat{U}}_{2}\left(\tau \right){\hat{U}}_{1}\left(\tau \right)\rho_{SR}{\hat{U}}_{1}^{†}\left(\tau \right){\hat{U}}_{2}^{†}\left(\tau \right)$ are the total state of the system and environment at time *t* and t+2τ, respectively. Since ${\mathrm{tr}}_{SR}\left\{{\hat{H}}_{tot}\left(s\right)\frac{\partial \rho_{SR}}{\partial s}\right\}=0$, the Formulation (6) is further reduced to

(7) $$\begin{array}{ccc} \Delta W & = & \int_{t}^{t+2\tau }{\langle \frac{\partial {\hat{H}}_{tot}\left(s\right)}{\partial s}\rangle }_{\rho_{SR}}ds\\ & = & \int_{t}^{t+\tau }\frac{\partial \lambda_{1}\left(s\right)}{\partial s}{\langle {\hat{H}}_{int}^{\left(1\right)}\rangle }_{\rho_{SR}}ds+\int_{t+\tau }^{t+2\tau }\frac{\partial \lambda_{2}\left(s\right)}{\partial s}{\langle {\hat{H}}_{int}^{\left(2\right)}\rangle }_{\rho_{SR}}ds\\ & \equiv & \sum_{i=1}^{2}\Delta W_{i}+\Delta W_{12}, \end{array}$$

where ${\langle \cdot \rangle }_{\varrho }\equiv \mathrm{tr}\left\{\cdot \varrho \right\}$ and the two components $\Delta W_{i}$ and $\Delta W_{12}$ constituting the total work ΔW can be formulated as

(8) $$\Delta W_{i}=\frac{\tau^{2}}{2}{\mathrm{tr}}_{SR}\left\{\left[{\hat{H}}_{int}^{\left(i\right)},\left[{\hat{H}}_{S_{i}}+{\hat{H}}_{R},{\hat{H}}_{int}^{\left(i\right)}\right]\right]\rho_{S}⊗\rho_{R}^{th}\right\}$$

and

(9) $$\Delta W_{12}=\tau^{2}{\mathrm{tr}}_{SR}\left\{\left[{\hat{H}}_{int}^{\left(1\right)},\left[{\hat{H}}_{S_{2}}+{\hat{H}}_{R},{\hat{H}}_{int}^{\left(2\right)}\right]\right]\rho_{S}⊗\rho_{R}^{th}\right\}.$$

We thus identify two types of work, i.e., the local one $\Delta W_{i}$ and nonlocal one $\Delta W_{12}$, which sustain the local collisions of $S_{i}$ with *R* and the cascaded collisions of $S_{1}$ and $S_{2}$ with *R*, respectively. Although $\Delta W_{i}$ can be formally derived by the collision model as if only $S_{i}$ exists in the absence of the other one, $\Delta W_{12}$ embodies the unique one-way influence of the cascaded model.

By means of Equations (7)–(9) and after taking the continuous time limit, we obtain the current of work as

(10) $$\dot{W}=\lim_{\tau \to 0}\frac{\Delta W}{\tau }=\sum_{i=1}^{2}{\dot{W}}_{i}+{\dot{W}}_{12}$$

with

(11) $${\dot{W}}_{i}=\frac{1}{2}{\mathrm{tr}}_{SR}\left\{\left[{\hat{V}}_{int}^{\left(i\right)},\left[{\hat{H}}_{S_{i}}+{\hat{H}}_{R},{\hat{V}}_{int}^{\left(i\right)}\right]\right]\rho_{S}⊗\rho_{R}^{th}\right\}$$

and

(12) $${\dot{W}}_{12}={\mathrm{tr}}_{SR}\left\{\left[{\hat{V}}_{int}^{\left(1\right)},\left[{\hat{H}}_{S_{2}}+{\hat{H}}_{R},{\hat{V}}_{int}^{\left(2\right)}\right]\right]\rho_{S}⊗\rho_{R}^{th}\right\}.$$

The heat in a collision can be unambiguously defined as the change in the energy of environment being of the form

(13) $$\begin{array}{ccc} \Delta Q & = & {\mathrm{tr}}_{SR}\left\{{\hat{H}}_{R}\left(\rho_{SR}^{\prime }-\rho_{SR}\right)\right\}\\ & = & \sum_{i=1}^{2}\Delta Q_{i}+\Delta Q_{12} \end{array}$$

with

(14) $$\Delta Q_{i}=\frac{\tau^{2}}{2}{\mathrm{tr}}_{SR}\left\{\left[{\hat{H}}_{int}^{\left(i\right)},\left[{\hat{H}}_{R},{\hat{H}}_{int}^{\left(i\right)}\right]\right]\rho_{S}⊗\rho_{R}^{th}\right\}$$

and

(15) $$\Delta Q_{12}=\tau^{2}{\mathrm{tr}}_{SR}\left\{\left[{\hat{H}}_{int}^{\left(1\right)},\left[{\hat{H}}_{R},{\hat{H}}_{int}^{\left(2\right)}\right]\right]\rho_{S}⊗\rho_{R}^{th}\right\}.$$

Obviously, the total heat ΔQ can be divided into local heat $\Delta Q_{i}$ and nonlocal heat $\Delta Q_{12}$, which are related to the local collision of $S_{i}$ with *R* and the nonlocal collision of $S_{1}$ and $S_{2}$ with *R*. Though $\Delta Q_{12}$ is generated by the collective collisions, it completely contributes to the heat of $S_{2}$ due to the unidirectional effect of the cascaded model [25].

By taking continuous time limit, the heat current can be derived as

(16) $$\dot{Q}=\lim_{\tau \to 0}\frac{\Delta Q}{\tau }=\sum_{i=1}^{2}{\dot{Q}}_{i}+{\dot{Q}}_{12}$$

where

(17) $${\dot{Q}}_{i}=\frac{1}{2}{\mathrm{tr}}_{SR}\left\{\left[{\hat{V}}_{int}^{\left(i\right)},\left[{\hat{H}}_{R},{\hat{V}}_{int}^{\left(i\right)}\right]\right]\rho_{S}⊗\rho_{R}^{th}\right\}$$

and

(18) $${\dot{Q}}_{12}={\mathrm{tr}}_{SR}\left\{\left[{\hat{V}}_{int}^{\left(1\right)},\left[{\hat{H}}_{R},{\hat{V}}_{int}^{\left(2\right)}\right]\right]\rho_{S}⊗\rho_{R}^{th}\right\}.$$

With the expression of internal energy of the system

(19) $$\begin{array}{ccc} \Delta U & = & {\mathrm{tr}}_{SR}\left\{{\hat{H}}_{S}\left(\rho_{SR}^{\prime }-\rho_{SR}\right)\right\}\\ & = & \frac{\tau^{2}}{2}\sum_{i}^{2}{\mathrm{tr}}_{SR}\left\{\left[{\hat{H}}_{int}^{\left(i\right)},\left[{\hat{H}}_{S_{i}},{\hat{H}}_{int}^{\left(i\right)}\right]\right]\rho_{S}⊗\rho_{R}^{th}\right\}\\ & & +\tau^{2}{\mathrm{tr}}_{SR}\left\{\left[{\hat{H}}_{int}^{\left(1\right)},\left[{\hat{H}}_{S_{2}},{\hat{H}}_{int}^{\left(2\right)}\right]\right]\rho_{S}⊗\rho_{R}^{th}\right\}, \end{array}$$

we can confirm that the derived thermodynamics quantities fulfill the first law of thermodynamics, i.e., ΔU=ΔW−ΔQ. Note that, by definition, the negative ΔQ means the heat flowing from the environment to the system.

## 4. Demonstration by Two-Level System

In order to demonstrate our results, we consider a fundamental configuration that both the system and ancillas are two-level systems (qubits) described by the free Hamiltonians ${\hat{H}}_{S_{i}}=\frac{\omega_{i}}{2}{\hat{\sigma }}_{S_{i}}^{z}$ and ${\hat{H}}_{R}=\frac{\omega_{R}}{2}{\hat{\sigma }}_{R}^{z}$, respectively, with $\omega_{i}$ ($\omega_{R}$) the frequency of subsystem $S_{i}$ (ancilla *R*). The interaction between $S_{i}$ and *R* is given as

(20) $$\begin{array}{ccc} {\hat{H}}_{int}^{\left(i\right)} & = & \frac{1}{\sqrt{\tau }}{\hat{V}}_{int}^{\left(i\right)}\\ & = & \frac{1}{\sqrt{\tau }}\left(J_{i}^{x}{\hat{\sigma }}_{S_{i}}^{x}⊗{\hat{\sigma }}_{R}^{x}+J_{i}^{y}{\hat{\sigma }}_{S_{i}}^{y}⊗{\hat{\sigma }}_{R}^{y}\right), \end{array}$$

where ${\hat{\sigma }}_{A}^{z}$, ${\hat{\sigma }}_{A}^{x}$ and ${\hat{\sigma }}_{A}^{y}$ are the usual Pauli operators acting on *A*.

The system’s dynamics is governed by the master Equation (3) with the dissipation terms being of the forms

(21) $$\begin{array}{ccc} L_{i}\left(\rho_{S}\right) & = & {\left(J_{i}^{x}\right)}^{2}\left({\hat{\sigma }}_{S_{i}}^{x}\rho_{S}{\hat{\sigma }}_{S_{i}}^{x}-\frac{1}{2}{\left[\rho_{S},{\hat{\sigma }}_{S_{i}}^{x}{\hat{\sigma }}_{S_{i}}^{x}\right]}_{+}\right)\\ & & +{\left(J_{i}^{y}\right)}^{2}\left({\hat{\sigma }}_{S_{i}}^{y}\rho_{S}{\hat{\sigma }}_{S_{i}}^{y}-\frac{1}{2}{\left[\rho_{S},{\hat{\sigma }}_{S_{i}}^{y}{\hat{\sigma }}_{S_{i}}^{y}\right]}_{+}\right)\\ & & -iJ_{i}^{x}J_{i}^{y}{\langle {\hat{\sigma }}_{R}^{z}\rangle }_{\rho_{R}}\left({\hat{\sigma }}_{S_{i}}^{x}\rho_{S}{\hat{\sigma }}_{S_{i}}^{y}-\frac{1}{2}{\left[\rho_{S},{\hat{\sigma }}_{S_{i}}^{y}{\hat{\sigma }}_{S_{i}}^{x}\right]}_{+}\right)\\ & & +iJ_{i}^{y}J_{i}^{x}{\langle {\hat{\sigma }}_{R}^{z}\rangle }_{\rho_{R}}\left({\hat{\sigma }}_{S_{i}}^{y}\rho_{S}{\hat{\sigma }}_{S_{i}}^{x}-\frac{1}{2}{\left[\rho_{S},{\hat{\sigma }}_{S_{i}}^{x}{\hat{\sigma }}_{S_{i}}^{y}\right]}_{+}\right) \end{array}$$

and

(22) $$\begin{array}{ccc} D_{12}\left(\rho_{S}\right) & = & i{\langle {\hat{\sigma }}_{R}^{z}\rangle }_{\rho_{R}}\left(J_{1}^{x}J_{2}^{y}\left[{\hat{\sigma }}_{S_{2}}^{y},\rho_{S}{\hat{\sigma }}_{S_{1}}^{x}\right]+J_{1}^{y}J_{2}^{x}\left[{\hat{\sigma }}_{S_{1}}^{y},\rho_{S}{\hat{\sigma }}_{S_{2}}^{x}\right]\right)\\ & & -i{\langle {\hat{\sigma }}_{R}^{z}\rangle }_{\rho_{R}}\left(J_{1}^{y}J_{2}^{x}\left[{\hat{\sigma }}_{S_{2}}^{x},\rho_{S}{\hat{\sigma }}_{S_{1}}^{y}\right]+J_{1}^{x}J_{2}^{y}\left[{\hat{\sigma }}_{S_{1}}^{x},\rho_{S}{\hat{\sigma }}_{S_{2}}^{y}\right]\right)\\ & & +J_{1}^{x}J_{2}^{x}\left[{\hat{\sigma }}_{S_{2}}^{x},\left[\rho_{S},{\hat{\sigma }}_{S_{1}}^{x}\right]\right]+J_{1}^{y}J_{2}^{y}\left[{\hat{\sigma }}_{S_{2}}^{y},\left[\rho_{S},{\hat{\sigma }}_{S_{1}}^{y}\right]\right]. \end{array}$$

The currents of local and nonlocal heat are derived as

(23) $${\dot{Q}}_{i}=\omega_{R}\left(2J_{i}^{x}J_{i}^{y}{\langle \sigma_{S_{i}}^{z}\rangle }_{\rho_{S}}-\left({\left(J_{i}^{x}\right)}^{2}+{\left(J_{i}^{y}\right)}^{2}\right){\langle \sigma_{R}^{z}\rangle }_{\rho_{R}}\right),$$

and

(24) $${\dot{Q}}_{12}=-2\omega_{R}{\langle \sigma_{R}^{z}\rangle }_{\rho_{R}}\left(J_{1}^{x}J_{2}^{x}{\langle \sigma_{S_{1}}^{x}\sigma_{S_{2}}^{x}\rangle }_{\rho_{S}}+J_{1}^{y}J_{2}^{y}{\langle \sigma_{S_{1}}^{y}\sigma_{S_{2}}^{y}\rangle }_{\rho_{S}}\right),$$

while the currents of local and nonlocal work are expressed as

(25) $$\begin{array}{ccc} {\dot{W}}_{i} & = & \omega_{i}\left(2J_{i}^{x}J_{i}^{y}{\langle \sigma_{R}^{z}\rangle }_{\rho_{R}}-\left({\left(J_{i}^{x}\right)}^{2}+{\left(J_{i}^{y}\right)}^{2}\right){\langle \sigma_{S_{i}}^{z}\rangle }_{\rho_{S}}\right)\\ & & +\omega_{R}\left(2J_{i}^{x}J_{i}^{y}{\langle \sigma_{S_{i}}^{z}\rangle }_{\rho_{S}}-\left({\left(J_{i}^{x}\right)}^{2}+{\left(J_{i}^{y}\right)}^{2}\right){\langle \sigma_{R}^{z}\rangle }_{\rho_{R}}\right) \end{array}$$

and

(26) $$\begin{array}{ccc} {\dot{W}}_{12} & = & 2J_{1}^{x}\left(\omega_{2}J_{2}^{y}-\omega_{R}J_{2}^{x}\right){\langle \sigma_{S_{1}}^{x}\sigma_{S_{2}}^{x}\rangle }_{\rho_{S}}{\langle \sigma_{R}^{z}\rangle }_{\rho_{R}}\\ & & +2J_{1}^{y}\left(\omega_{2}J_{2}^{x}-\omega_{R}J_{2}^{y}\right){\langle \sigma_{S_{1}}^{y}\sigma_{S_{2}}^{y}\rangle }_{\rho_{S}}{\langle \sigma_{R}^{z}\rangle }_{\rho_{R}}. \end{array}$$

From the expressions of local currents of heat and work, i.e., ${\dot{Q}}_{i}$ in Equation (23) and ${\dot{W}}_{i}$ in (25), we can see that the prior interaction of $S_{1}-R$ will exert a one-way influence on the local currents of $S_{2}$ if any, through affecting the reduced state of $S_{2}$ and eventually the term ${\langle \sigma_{S_{2}}^{z}\rangle }_{\rho_{S}}$. The condition for the disappearance of ${\dot{Q}}_{i}$ is $J_{i}^{x}=J_{i}^{y}$ and meanwhile $\omega_{i}\beta_{i}=\omega_{R}\beta_{R}$ (i.e., ${\langle \sigma_{S_{i}}^{z}\rangle }_{\rho_{S}}={\langle \sigma_{R}^{z}\rangle }_{\rho_{R}}$) with $\beta_{i}$ the steady-state inverse temperature of $S_{i}$. As for ${\dot{W}}_{i}$, it will vanish when the interaction of $S_{i}-R$ satisfies strict energy conservation, namely, $\omega_{i}=\omega_{R}$ and $J_{i}^{x}=J_{i}^{y}$. From Equations (24) and (26), we can see that the nonlocal currents of heat and work are closely related to the establishment of correlations between $S_{1}$ and $S_{2}$ in terms of ${\langle \sigma_{S_{1}}^{x(y)}\sigma_{S_{2}}^{x(y)}\rangle }_{\rho_{S}}$. Moreover, the formulation of (26) indicates that the nonlocal work current ${\dot{W}}_{12}$ is bound to vanish if $\omega_{2}=\omega_{R}$ and $J_{2}^{x}=J_{2}^{y}$, i.e., ${\dot{W}}_{2}=0$; however, this does not imply that ${\dot{W}}_{12}$ is only determined by the interaction of $S_{2}-R$ since even when ${\dot{W}}_{2}\neq 0$, a finite nonzero nonlocal work current ${\dot{W}}_{12}$ requires the existence of correlations of $S_{1}$ and $S_{2}$.

In the following, we shall demonstrate the behaviors of steady-state currents of heat and work in detail by considering whether the interactions of $S_{1}-R$ and $S_{2}-R$ satisfy energy conservation or not. A simple situation is that both interactions of $S_{1}-R$ and $S_{2}-R$ are energy preserving in the sense that $J_{i}^{x}=J_{i}^{y}$ and $\omega_{i}=\omega_{R}$ for i=1,2. The system will then relax towards the equilibrium stationary state (ESS) [25]

(27) $$\rho_{S}\left(\infty \right)=\frac{e^{-\beta_{R}{\hat{H}}_{S_{1}}}}{Z_{1}}⊗\frac{e^{-\beta_{R}{\hat{H}}_{S_{2}}}}{Z_{2}},$$

so that both the currents of heat and work vanish in the stationary regime. Albeit the steady-state feature in this case is trivial, the memory effects on the dynamics of heat current exhibit rich phenomenons as discussed in Ref. [25].

If the interaction of $S_{1}-R$ is energy preserving with $J_{1}^{x}=J_{1}^{y}$ and $\omega_{1}=\omega_{R}$, we find that apart from the vanishing currents of work and heat regarding $S_{1}$, i.e., ${\dot{W}}_{1}={\dot{Q}}_{1}=0$, the nonlocal currents also become zero, i.e., ${\dot{W}}_{12}={\dot{Q}}_{12}=0$ as no correlations can be constructed in this case. Moreover, the prior interaction of $S_{1}-R$ does not exert any influences on $S_{2}$ in other words, the steady-state currents of $S_{2}$ are not influenced by $S_{1}$ manifesting behaviors as if the interaction of $S_{1}-R$ does not exist; therefore, only the energy conservation of $S_{1}-R$ does not hold, as it has an impact on $S_{2}$, which is to be discussed in the following.

### 4.1. The Interaction of $S_{2}-R$ Is Energy-Preserving

We first consider the situation that the interaction of $S_{1}-R$ is not energy-conserving, while the interaction of $S_{2}-R$ still satisfies strict energy conservation with $J_{2}^{x}=J_{2}^{y}$ and $\omega_{2}=\omega_{R}$. In this case, the steady-state correlation between $S_{1}$ and $S_{2}$ can be established, which makes $S_{2}$ fail to reach ESS although the interaction of $S_{2}-R$ is energy-conserving. Eventually, both $S_{1}$ and $S_{2}$ reach NESS with effective inverse temperatures $\beta_{eff1}$ and $\beta_{eff2}$ that could deviate from $\beta_{R}$ of the environment to different extents. The effective temperature of $S_{i}$ can be defined as $T_{effi}=1/\beta_{effi}=\omega_{i}/\ln\left(p_{i}^{g}/p_{i}^{e}\right)$ with $p_{i}^{g}\left(p_{i}^{e}\right)$ being the stationary population of the ground (excited) state of the subsystem $S_{i}$. As shown in Equations (25) and (26), the strict energy conservation of the interaction of $S_{2}-R$ means ${\dot{W}}_{2}={\dot{W}}_{12}=0$, namely, no work is provided through the interaction of $S_{2}-R$; therefore, the NESS of $S_{2}$, alternatively speaking, the total NESS of $S_{1}$ and $S_{2}$ is sustained by the work invested via $S_{1}$. We also note that in this case ${\dot{Q}}_{2}\neq 0$; nevertheless, the nonlocal heat current ${\dot{Q}}_{12}=-{\dot{Q}}_{2}$ so that the total heat current associated with $S_{2}$ stays zero, being consistent with the vanishing ${\dot{W}}_{2}$ and ${\dot{W}}_{12}$.

**Figure 2:**
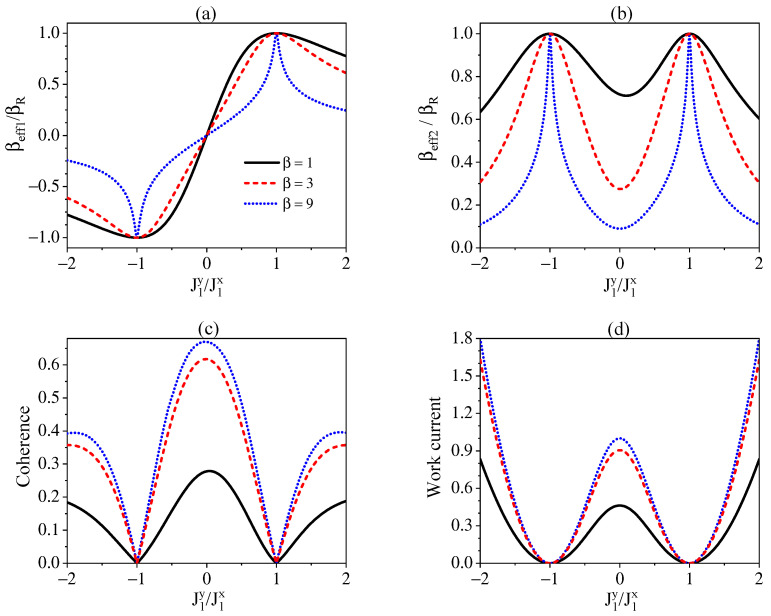
The ratios of effective inverse temperatures $\beta_{eff1}$ and $\beta_{eff2}$ of $S_{1}$ (**a**) and $S_{2}$ (**b**) to that of environment $\beta_{R}$, the coherence of the system (**c**), and the work current (**d**) as a function of $J_{1}^{y}/J_{1}^{x}$ for different $\beta_{R}$. We set $J_{2}^{y}=J_{2}^{x}=\omega$ and $\omega_{1}=\omega_{2}=\omega_{R}=\omega$.

In Figure 2a,b, we illustrate deviations of effective inverse temperatures of $S_{1}$ and $S_{2}$ from that of the environment in terms of the ratios $\beta_{eff1}/\beta_{R}$ and $\beta_{eff2}/\beta_{R}$. A comparison between $\beta_{eff1}/\beta_{R}$ and $\beta_{eff2}/\beta_{R}$ in Figure 2a,b shows that $S_{1}$ can reach ESS with $\beta_{eff1}=\beta_{R}$ only at the point of $J_{1}^{x}=J_{1}^{y}$ and the negative temperatures are achieved when $J_{1}^{y}/J_{1}^{x}<0$, whereas $S_{2}$ can arrive at ESS with $\beta_{eff2}=\beta_{R}$ at the two points of $J_{1}^{x}=\pm J_{1}^{y}$. The nonconservation of energy for $S_{1}-R$ induces steady-state correlations between $S_{1}$ and $S_{2}$, which is quantified by the $l_{1}-$ norm of coherence defined as $C=\sum_{l\neq m}\left|\rho_{lm}\right|$ with $\rho_{lm}$ the matrix elements of density operator ρ [80]. Apart from the effective temperature, the existence of correlation is also a signature of the system reaching NESS. The coherence of $S_{1}$ and $S_{2}$ as a function of $J_{1}^{y}/J_{1}^{x}$ is demonstrated in Figure 2c. By comparing Figure 2a, Figure 2b, and Figure 2c, we observe that the farther the individual subsystem $S_{i}$ (i=1,2) deviates from equilibrium, i.e., the smaller the ratios $|\beta_{effi}/\beta |<1$, the larger the coherence. We also note that the lower the temperature of environment, the larger the coherence. The NESS of the total system, which is characterized now by both the effective temperatures of individual subsystems and the coherence, should be sustained by the work current supplied by an external agent. In Figure 2d, our displays of the work current show that the work cost is consistent with the extent of the system deviating from the ESS, namely, the smaller the ratio $|\beta_{effi}/\beta_{R}|$ and the larger the coherence, the more the work current.

### 4.2. Both the Interactions of $S_{1}-R$ and $S_{2}-R$ Are Not Energy-Preserving

**Figure 3:**
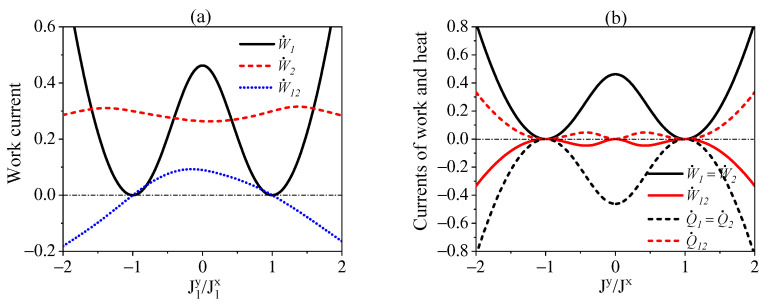
(**a**) The work currents against $J_{1}^{y}/J_{1}^{x}$ for $J_{2}^{x}=0.6\omega$, $J_{2}^{y}=1.2\omega$. (**b**) The currents of heat and work against $J^{y}/J^{x}$ for the symmetric couplings of $S_{1}-R$ and $S_{2}-R$ with $J_{2}^{x}=J_{1}^{x}=J^{x}$, $J_{2}^{y}=J_{1}^{y}=J^{y}$. We set $\omega_{1}=\omega_{2}=\omega_{R}=\omega$ and $\beta_{R}=\omega$.

We finally consider the scenario where both the interactions of $S_{1}-R$ and $S_{2}-R$ are not energy-preserving, for which the nonlocal steady-state currents of work and heat might appear as indicated in Equations (24) and (26). Here, we are interested in the one-way influences of the interaction of $S_{1}-R$ on the steady-state currents of $S_{2}$. For this purpose, we demonstrate in Figure 3a the local and nonlocal currents regarding both $S_{1}$ and $S_{2}$ against the interaction of $S_{1}-R$ in terms of $J_{1}^{y}/J_{1}^{x}$. We can clearly observe the variations of ${\dot{W}}_{2}$ and ${\dot{W}}_{12}$ with respect to $J_{1}^{y}/J_{1}^{x}$ implying influences of the interaction of $S_{1}-R$ on the work currents of $S_{2}$. Moreover, the nonlocal current ${\dot{W}}_{12}$ can be transformed between positive and negative values, which means that the interaction of $S_{1}-R$ is able to control the direction of ${\dot{W}}_{12}$. The currents of heat exhibit similar behaviors to that of the work, which is not shown here. We also note that the symmetric interactions of $S_{1}-R$ and $S_{2}-R$ with $J_{1}^{x}=J_{2}^{x}=J^{x}$ and $J_{1}^{y}=J_{2}^{y}=J^{y}$ can lead to ${\dot{W}}_{1}={\dot{W}}_{2}$ and ${\dot{Q}}_{1}={\dot{Q}}_{2}$, as demonstrate in Figure 3b. This implies that the local currents of work and heat for the symmetric interactions are the same as that would be obtained in the independent interactions for each subsystem with the environment. Since in this case the nonlocal current of work ${\dot{W}}_{12}$ always take opposite directions to the local ones, the total cost of work in the presence of cascaded interactions, i.e., ${\dot{W}}_{tot}^{cas}={\dot{W}}_{1}+{\dot{W}}_{2}+{\dot{W}}_{12}$, are always less than that with only independent interactions, i.e., ${\dot{W}}_{tot}^{ind}={\dot{W}}_{1}+{\dot{W}}_{2}$, in the sense that ${\dot{W}}_{tot}^{cas}<{\dot{W}}_{tot}^{ind}$.

## 5. The Extractable Work in Cascaded Model

From above discussions, we recognize that steady-state coherence of the system can be available due to cascaded interactions of subsystems with the common environment, exhibiting striking contrast to the situation of independent interactions where no any coherence can be generated; therefore, it is interesting to study the role of coherence in extracting useful work from the system. The maximum work that can be extracted from a quantum system via cyclic and unitary operations is quantified by the so-called ergotropy [81], which is given as the difference between the energy of the initial state and that of the final state with the minimum average energy through all possible unitary operations. For a quantum system described by Hamiltonian $\hat{H}=\sum_{k=1}^{d}\varepsilon_{k}|\varepsilon_{k}\rangle \langle \varepsilon_{k}|$ and density operator $\rho =\sum_{l=1}^{d}r_{l}|r_{l}\rangle \langle r_{l}|$ such that $\varepsilon_{k}\leq \varepsilon_{k+1}$ and $r_{l}\geq r_{l+1}$, the ergotropy can be defined as

(28) $$E\left(\rho \right)=\mathrm{Tr}\left\{\hat{H}\rho \right\}-\mathrm{Tr}\left\{\hat{H}{\hat{U}}_{min}\rho {\hat{U}}_{min}^{†}\right\}=\mathrm{Tr}\left\{\hat{H}\left(\rho -P_{\rho }\right)\right\}$$

where $P_{\rho }=\sum_{l}r_{l}|\varepsilon_{l}\rangle \langle \varepsilon_{l}|$ is called the passive state. By plugging the explicit form of $P_{\rho }$ in (28), we obtain the well-known expression of ergotropy as

(29) $$E\left(\rho \right)=\sum_{k,l}r_{l}\varepsilon_{k}\left({\left|\langle r_{l}|\varepsilon_{k}\rangle \right|}^{2}-\delta_{kl}\right).$$

**Figure 4:**
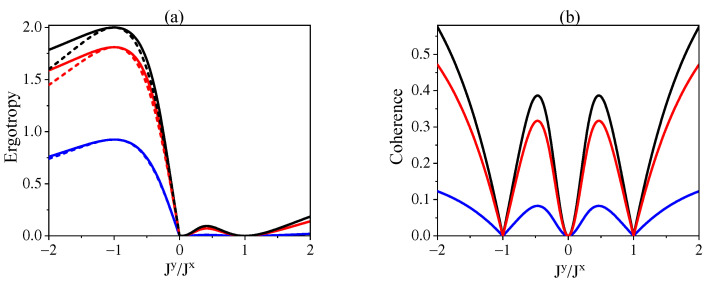
(**a**) The ergotropy for cascaded interactions (solid lines) and independent interactions (dashed lines) of $S_{1}$ and $S_{2}$ with *R* against $J^{y}/J^{x}$. (**b**) The corresponding coherence of the system under cascaded interactions. We set symmetric couplings of $S_{1}-R$ and $S_{2}-R$ with $J_{1}^{x}=J_{2}^{x}=J^{x}$ and $J_{1}^{y}=J_{2}^{y}=J^{y}$. The other parameters are set as $\beta_{R}=9\omega$ (black lines), $\beta_{R}=3\omega$ (red lines), $\beta_{R}=\omega$ (blue lines), and $\omega_{1}=\omega_{2}=\omega_{R}=\omega$.

Here, we make a comparison for the ergotropy in two configurations, namely, the cascaded interactions and the independent interactions of $S_{1}$ and $S_{2}$ with *R*. For this purpose, we consider the symmetric interactions of $S_{1}$ and $S_{2}$ with *R* with $J_{1}^{x}=J_{2}^{x}=J^{x}$ and $J_{1}^{y}=J_{2}^{y}=J^{y}$. Moreover, for the independent interactions, we define the total ergotropy as $E^{ind}\left(\rho \right)=E\left(\rho_{S_{1}}\right)+E\left(\rho_{S_{2}}\right)$ with $\rho_{S_{i}}$(i=1,2) the state of $S_{i}$. The behavior of ergotropy is illustrated in Figure 4a for different temperatures of the environment. We observe that at the region of $J^{y}/J^{x}<0$ a finite nonzero ergotropy appears for both cascaded and independent interactions of $S_{1}-R$ and $S_{2}-R$ with magnitude of the former case is always larger than that of the latter case. By contrast, for the region of $J^{y}/J^{x}>0$, the ergotropy retains a nonzero value only for the cascaded interactions. The results can be understood by recalling that the ergotropy under independent interactions is contributed totally by the population inversions of the subsystems with negative effective temperatures, which can occur only in the region of $J^{y}/J^{x}<0$ (cf. Figure 2a). By contrast, the ergotropy under cascaded interactions is contributed by both the population inversions of individual subsystems and the coherence of the total system. As a result, the ergotropy of cascaded interactions in the region of $J^{y}/J^{x}<0$ is always larger than that of independent interactions and arises also in the region of $J^{y}/J^{x}>0$ where the correlations can be established. To have a visualized picture, we plot the coherence of system under the cascaded interaction in Figure 4b. A comparison between Figure 4a and Figure 4b actually confirms our explanations according to the following observations. At the point of $J^{y}/J^{x}=-1$, the coherence becomes zero so that the ergotropy in cascaded and independent interactions coincide with each other. In the region of $J^{y}/J^{x}>0$, the ergotropy is completely contributed by the coherence and the larger the coherence with the lower temperature of the environment, the larger the ergotropy. At the point of $J^{y}/J^{x}=1$, the zero coherence leads to vanishing ergotropy. Moreover, the larger the coherence-based ergotropy, the greater the difference for the ergotropy in these two configurations, as shown in the region of $J^{y}/J^{x}<0$.

## 6. Conclusions

In conclusion, by virtue of collision model, we have studied the thermodynamics of a bipartite system with subsystems $S_{1}$ and $S_{2}$ interacting with an environment *R* in the cascaded fashion, namely, the environment *R* collides/interacts firstly with $S_{1}$ and subsequently with $S_{2}$. Thanks to the framework of the collision model, we have constructed the general forms of work and heat of the system in both discrete steps and continuous time limit from their most fundamental definitions. The constructed formulations allow us to identify the local and nonlocal components of the work and heat and discuss their features due to the cascaded interactions. Focusing on the two-qubit system and concrete form of system–environment interactions, we have demonstrated our results and revealed the necessary condition for the unidirectional influence of the prior interaction of $S_{1}-R$ to the thermodynamics of $S_{2}$ in the steady-state regime. It turns out that the one-way influence occurs only when the interaction of $S_{1}-R$ does not fulfill energy conservation. With the influence of prior interaction, subsystem $S_{2}$ cannot arrive at ESS even when the interaction of $S_{2}-R$ satisfies strict energy conservation. In case the interaction of $S_{2}-R$ does not satisfy energy conservation at the same time, the interaction of $S_{1}-R$ will have an impact on both the local work regarding $S_{2}$ and nonlocal work. We also show that the steady-state coherence generated by the cascaded interaction is a useful resource in extracting work in terms of ergotropy from the system. Our results thus reveal the unique thermodynamics features in the cascaded model and particularly provide a possible way to achieve the one-way control on the thermodynamics process in the steady-state regime.

## Data Availability

Not applicable.
